# Cell iron status influences macrophage polarization

**DOI:** 10.1371/journal.pone.0196921

**Published:** 2018-05-17

**Authors:** Rafiou Agoro, Meriem Taleb, Valerie F. J. Quesniaux, Catherine Mura

**Affiliations:** 1 Experimental and Molecular Immunology and Neurogenetics, UMR 7355 CNRS, Orléans, France; 2 University of Orléans, Orléans, France; Universitatsklinikum Freiburg, GERMANY

## Abstract

Macrophages play crucial roles in innate immune response and in the priming of adaptive immunity, and are characterized by their phenotypic heterogeneity and plasticity. Reprogramming intracellular metabolism in response to microenvironmental signals is required for M1/M2 macrophage polarization and function. Here we assessed the influence of iron on the polarization of the immune response *in vivo* and *in vitro*. Iron-enriched diet increased M2 marker *Arg1* and *Ym1* expression in liver and peritoneal macrophages, while iron deficiency decreased *Arg1* expression. Under LPS-induced inflammatory conditions, low iron diet exacerbated the proinflammatory response, while the IL-12/IL-10 balance decreased with iron-rich diet, thus polarizing toward type 2 response. Indeed, *in vitro* macrophage iron loading reduced the basal percentage of cells expressing M1 co-stimulatory CD86 and MHC-II molecules. Further, iron loading of macrophages prevented the pro-inflammatory response induced by LPS through reduction of NF-κB p65 nuclear translocation with decreased iNOS, IL-1β, IL-6, IL-12 and TNFα expression. The increase of intracellular iron also reduced LPS-induced *hepcidin* gene expression and abolished *ferroportin* down-regulation in macrophages, in line with macrophage polarization. Thus, iron modulates the inflammatory response outcome, as elevated iron levels increased M2 phenotype and negatively regulated M1 proinflammatory LPS-induced response.

## Introduction

Macrophages play a central role in both immunity and tissue homeostasis, protecting the organism from infection as well as completing essential tissue-specific functions. Indeed, macrophage immune functions include pathogen recognition, antigen processing, inflammation, phagocytic clearance, immune regulation as well as resolution of immune responses and tissue repair [1]. Macrophages also play an essential role in iron homeostasis by recycling iron, a crucial transition metal for living organisms. Indeed, in mammals, most of the iron used for hemoglobin production is recycled after phagocytosis of senescent red blood cells by macrophages, with a limited intake of dietary iron absorbed through enterocytes. Clearly, interactions between iron homeostasis and immune functions of macrophages have been reported. Systemic iron balance is maintained via hepcidin hormone and ferroportin cellular iron exporter [2–6] and macrophage polarization may affect their expression [7–9]. Indeed, *hepcidin 1* upregulation, controlled at the transcriptional level, is exacerbated by M1 polarization while *ferroportin* transcription is influenced by M2 polarisation [10, 11]. Conversely, several publications indicated a change in inflammatory response according to the iron status, but conflicting results have been reported [12–15]. Here, we questioned the influence of iron on macrophage polarization.

Macrophages represent a heterogeneous cell population with a dynamic spectrum of functional states from pro-inflammatory M1 to anti-inflammatory/immunoregulatory alternatively activated M2 macrophages, exhibiting marked differences in the gene expression signatures and effector functions [1, 16, 17]. Activated M1 macrophages stimulate type 1 T helper (Th1) adaptive responses, while alternatively activated M2 macrophages, which have been divided into several subsets depending on the differentiation/activation signals encountered, stimulate the Th2 adaptive response essential for humoral-mediated immunity, immunoregulatory and anti-inflammatory functionalities, pro-fibrotic and repair activities, parasite resistance and tumor promotion [1, 18]. Distinct functional phenotypes of macrophage M1 and M2 depend on a coordinated expression of various modulators promoting opposed functions. Indeed, M1 are characterized by high levels of IL-12 pro-inflammatory and low IL-10 anti-inflammatory cytokine expression (IL-12^hi^/IL-10^lo^), while M2 are IL-12^lo^/IL-10^hi^. M1 and M2 are also characterized by the expression of alternative L-arginine metabolizing enzyme activities, iNos and Arg1, respectively, producing NO and ornithine that correlate to either killing and repairing functions [1, 16, 17, 19–24]. The release of IFNγ, TNF-α, IL-1β, chemokines and proteases, together with production of reactive oxygen species (ROS) [1, 16, 17, 19–24]. is also a marked characteristic of M1, while M2 express other molecules including chitinase family proteins, and mannose receptor C-type 1 CD206 [16, 21–25]. Macrophage functions result from differential activation of transcription factors such as STAT, IRF, and NF-κB [17, 26–28] and metabolic status which contribute to the control of macrophage activation [29–31]. These pathways are activated by various stimuli, including pathogen associated molecular patterns (PAMPs), as well as endogenous cytokines, chemokines, nutrients and danger signals [32–34, 26]. The field of macrophage polarization expanded, both in terms of the range of inducing signals, which include tissue microenvironment and metabolites, and their resulting specificity and functional diversity [16, 19, 20, 35], and we addressed the role of iron, as a main cell growth factor, in macrophage polarization.

The couple hepcidin and ferroportin regulate systemic iron balance [2–6]. Hepcidin is mainly synthesized from hepatocytes and upregulated during iron overload through Bmp6-Smad1/5/8/4 pathway activation. Hepcidin is released in blood circulation and binds to the cellular iron exporter ferroportin, leading to its internalization and degradation [2, 6, 36, 37]. Thus, the systemic signal given by hepcidin decreases the transfer of iron from the enterocytes to the rest of the body by ferroportin and iron recycling from macrophages. Splenic macrophages and liver Kupffer cells contribute to erythrophagocytosis and influence the daily turn-over of iron by increasing *ferroportin* mRNA transcription [38, 39]. In isolated macrophages, while *hepcidin 1* gene is not upregulated by increased iron levels, the expression of *ferroportin* mRNA is upregulated upon iron cell loading and can be downregulated after addition of exogenous hepcidin [40, 41]. The expression of *hepcidin 1* and *ferroportin* genes is also altered upon inflammation. Liver and macrophage *hepcidin 1* expression is upregulated through Smad1/5/8/4 and Stat3 pathways in response to inflammation, while *ferroportin* transcription is downregulated at the transcriptional level, independently of hepcidin, both leading to hypoferremia [42–47].

Here we addressed the influence of iron on innate immune phenotype and macrophage polarization *in vivo* and *in vitro*, and we investigate the cross-talk between inflammation and iron balance regulation. We show that, while iron depletion reduces M2 markers, an iron-rich status promotes M2-like phenotype and reduces type 1 immune response markers, impairing LPS-induced pro-inflammatory cytokine expression, and this is accompanied by a differential regulation of *hepcidin 1* and *ferroportin* expression under inflammatory conditions.

## Materials and methods

### Ethics statement

All animal experimental protocols complied with the French ethical and animal experiments regulations (see Charte Nationale, Code Rural R 214–122, 214–124 and European Union Directive 86/609/EEC) and were approved by the “Ethics Committee for Animal Experimentation of CNRS Campus Orleans” (CCO), registered (N°3) by the French National Committee of Ethical Reflexion for Animal Experimentation, under N° CLE CCO 2015–1085.

### *In vivo* experiments

C57BL/6 wild-type mice (Janvier Labs, Le Genest-Saint-Isle, France) housed in the Transgenose Institute animal facility (TAAM, CNRS UPS44, Orleans) were fed with either iron-rich diet (25000 mg of iron carbonyl/kg) for 3 days, iron-deficient diet (less than 20 mg iron carbonyl/kg) for 14 days, or iron-replete diet as control diet (280 mg iron carbonyl/kg, R03 from Scientific Animal Food & Engineering; Augy, France). For some experiments mice received 0.2 g/kg of iron-dextran by peritoneal injection 48 hours before necropsy. Mice were observed daily and monitored for signs of distress including ruffled fur, immobility or hunching, and body weight loss. Acute inflammation was induced by a single intraperitoneal injection of lipopolysaccharide (LPS *E*. *coli* 055:B5, Sigma-Aldrich, St Louis, MO, USA; 50 μg/kg), and equivalent volume of sterile saline solution (0.9% NaCl) was used as vehicle control. After 4 hours, mice were bled under isoflurane anesthesia, then killed with carbon dioxide inhalation, liver and spleen removed, and peritoneal exudate cells harvested as a source of peritoneal macrophages.

### Histology of iron staining

Liver and spleen tissues were fixed in 4% phosphate buffered formalin, paraffin-embedded, and 5 μm sections prepared. DAB-enhanced Perl’s staining was performed on paraffin sections treated successively with 5% potassium ferrocyanide /4% HCl, 0.01M sodium azide /0.3% H_2_O_2_ in methanol, 0.025% 3,3’-diaminobenzidine tetrahydrochloride (DAB). Sections were counterstained with Nuclear Fast Red (Sigma). Iron distribution was determined by light microscopy.

### Measurement of hepatic iron content

Hepatic iron content (HIC) was measured from dried liver samples dissolved in nitric acid:sulfuric acid (1:1) at 90°C, then after adding 1 mL of H_2_O_2_, an equivalent volume of sample and 10 mM ferrozine / 32.6 mM ascorbic acid / 50 mM Tris HCl pH4 was incubated 30 min before reading at 560 nm. The values were expressed as ng of iron per mg of dry tissue.

### Hematology and blood markers

Blood was obtained from retro-orbital plexus under isoflurane anesthesia. Serum iron and unbound iron binding capacity was measured by using ferrozine as described by Goodwin *et al*. [48]. Transferrin saturation was calculated as follows: serum iron/ (serum iron + unbound iron binding capacity) × 100%. Red blood cell count (RBC), mean corpuscular volume (MCV), hematocrit (Ht), hemoglobin (Hb), were measured from EDTA-plasma with MS9-5V Melet schloesing apparatus.

### Cell culture

Bone marrow cells from C57BL/6 wild-type were isolated from femurs and differentiated into macrophages after culturing at 10^6^ cells/ml for 10 days in DMEM (Sigma-Aldrich) supplemented with 10 mM glutamine, 25 mM Hepes, 100 U/ml penicillin and 100 μg/ml streptomycin (all from Gibco-Thermo Fisher Scientific Inc, Waltham, MA, USA), plus 20% horse serum (HyClone Laboratories, Utah, USA) and 30% L929 cell-conditioned medium as a source of M-CSF [49]. When indicated cells were treated with 100 ng/mL LPS (*E*. *coli* 055:B5), ferric ammonium citrate (FAC, 10, 50 or 100 μM, Sigma-Aldrich), IFN-γ (100 U/mL) or IL-4 (10 ng/mL, R&D Systems Inc, Minneapolis, MN, USA). Cell viability was assessed by cell membrane exclusion of 7-Aminoactinomycin D (7-AAD) dye binding to DNA, followed by viable/dead cells analysis by flow cytometry (488/650 nm).

### Labile cell iron and reactive oxygen species measurement

Labile cell iron and intracellular ROS were measured by using calcein acetoxymethyl (calcein-AM) and 2’,7’-dichlorodihydrofluorescein diacetate (H2DCFDA), respectively. Bone marrow-derived macrophages plated in 96-well microplate (OptiPlate, PerkinElmer-Thermo Fisher Scientific Inc, Waltham, MA, USA) overnight were washed with PBS and incubated either 5 min with 5 μM of calcein acetoxymethyl (calcein-AM, Life Technologies-Thermo Fisher Scientific Inc, Waltham, MA, USA) or 30 min with 5 μM of fluorogenic probe 2’,7’-dichlorodihydrofluorescein diacetate (H2DCFDA, Life Technologies) for labile cell iron and intracellular ROS measurement, respectively. After wash, cells were further incubated with ferric ammonium citrate (FAC, Sigma-Aldrich) as indicated and fluorescence intensity was measured using fluorescent plate reader (Clariostar, BMG Labtech, Ortenberg, Germany). For calcein data are given as the delta (Δ) of the fluorescence to initial fluorescence.

### NF-κB staining by immunofluorescence analysis

Bone marrow derived macrophages (BMDM) plated on slides were incubated with or without FAC (Sigma-Aldrich) overnight followed by LPS for 30 min. BMDM were then fixed with paraformaldehyde 4% (Sigma Aldrich) for 10 minutes and, after blocking with Tris 1M pH7.6/NaCl 0.73M containing 10% FCS (HyClone), 1% BSA (Eurobio, Courtaboeuf, France) and 0.2% Triton X-100 (Sigma-Aldrich) for 45 minutes, were incubated with the anti-NF-κB p65 antibody (R&D systems Inc) overnight at 4°C. Subsequently, cells were washed with Tris 1M pH7.6/NaCl 0.73M and incubated with the secondary antibody conjugated with Alexa Fluor 488 (abcam Cambridge, UK) for 2 hours and counterstained with 4’,6-Diamidino-2-Phenylindole Dihydrochloride (DAPI, Sigma Aldrich). Cells were washed and photographs were acquired using Axio OBSERVER Z1 microscope plan-Apochromat 63x/1,40 Oil (Carl ZEISS, Oberkochen, Germany) and analyzed with ImageJ software.

### Cytokine determination in cell culture medium

After stimulation, cell supernatants were harvested to quantify IL-12p40, IL-1β, IL-6 and TNF-α concentrations using enzyme-linked immunosorbent assays (ELISA; Duoset, R&D).

### Nitric oxide production

The production of nitric oxide (NO) was measured in culture supernatants by colorimetric Griess assay for nitrites/nitrates [50]. An equal volume of culture supernatant sample and Griess reagent 0.5% sulfanilamide / 0.05% N-1-napthylethylenediamine dihydrochloride / 2.5% phosphoric acid) (all chemicals were from Sigma-Aldrich) was mixed and incubated for 30 min. A standard curve was established with NaNO_2_ and absorbance was measured at 562 nm.

### Flow cytometry analysis

Macrophages were plated in Petri dish at 5×10^6^ cells per dish. Cells were then stimulated with FAC, IFN-γ or IL-4 as indicated. Cells were washed and stained with 7-aminoactinomycin D (7-AAD), anti-CD86 PE (BD Pharmingen, San Diego, CA, USA), anti-IA/IE APC (BD Pharmingen), anti-CD206 (Bio Legend, San Diego, CA, USA), or anti-F4/80 (eBioscience, San Diego, CA, USA). Cells were then detached with cold PBS and analyzed by flow cytometry.

### Quantitative PCR analysis

Total RNA from liver and spleen homogenates or cells was isolated using TRI Reagent (Sigma-Aldrich) and reverse transcription was performed with Promega synthesis system (Promega, Madison, WI, USA) according to the manufacturer’s instructions. Quantitative PCR was performed with QuantiTect SYBR Green PCR system (Qiagen, Hilden, Germany) on Stratagene Mx3005P (Agilent Technologies, Santa Clara, CA, USA) by using *hepcidin 1* forward and reverse primers: 5’-CCTATCTCCATCAACAGATG-3’ and 5’-TGCAACAGATACCACACTG-3’, *ferroportin* forward and reverse primers: 5’-CTCTGTCAGCCTGCTGTTTG-3’ and 5’-TCAGGATTTGGGGCCAAGATG -3’, other primers were Quantitect primers (Qiagen, Hilden, Germany). The mRNA levels were normalized to the housekeeping gene *Gapdh* in the same RT sample and relative transcript expression of genes was given as Δ*C*t = *C*_t target_−*C*_t Gapdh_. Fold change of genes expression compared with untreated mice was determined as 2^−ΔΔ*C*t^ values (ΔΔ*C*t = Δ*C*_t treated_−Δ*C*_t control_).

### Statistical analysis

Student’s *t*-test and one-way Anova test were used to determine the significance of the data by using GraphPrism software (version 5.04 for Windows, GraphPad Software, La Jolla, CA, USA). Significant levels were indicated as *, p < 0.05, **, p < 0.01, ***, p < 0.001.

## Results

### Host iron status controls M2 marker expression in liver and peritoneal macrophages

**Fig 1:**
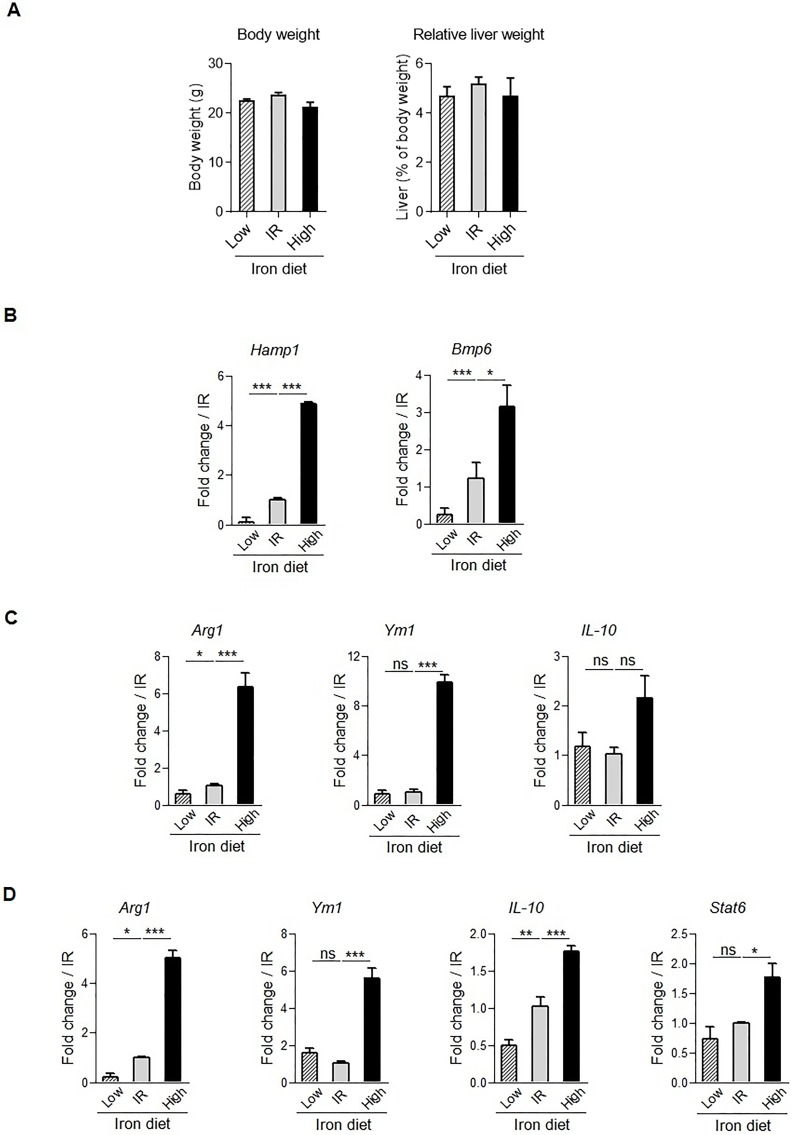
Association of iron status and immune response marker expression in liver and peritoneal macrophages. C57BL/6 wild type mice were fed with iron replete diet (IR), deficient diet for 14 days (Low) or iron-rich diet for 3 days (High). (**A**) Body weight and relative liver weights are indicated. Liver homogenates **(B, C),** and peritoneal macrophages (**D**) gene expression was then analyzed using quantitative PCR analysis. Data are expressed as mRNA fold change relative to control mice fed with IR diet. Data are from two independent experiments and presented as mean ± SD (n = 6 mice per group). ns, non significant; * p≤ 0.05; ** p≤ 0.01; *** p≤ 0.001.

Adult wild type C57BL/6 mice received specific iron-containing diet to induce either dietary iron loading or dietary iron deficiency. These diets had little effect on the overall body weight and relative liver weights (Fig 1A). Liver gene expression of *hepcidin* anti-microbial peptide (*Hamp1)* and bone morphogenic protein (*Bmp6*), the key iron-responsive inducer of *Hamp1* transcription, were then assessed to confirm the change of iron metabolism parameters induced by the specific diets. As expected, *Hamp1* and *Bmp6* mRNA expression in liver homogenates was increased with iron-rich diet and decreased with iron-deficient diet (Fig 1B), confirming their value as biomarkers of iron status. We next determined whether the modification of dietary iron and liver iron genes level was correlated with markers of immune response. Interestingly, in liver the expression of type 2 immune response markers *Arginase 1* (*Arg1*) and *Chitinase-like 3 (Ym1)* was increased after high iron diet correlating with a trend of increase expression of *IL-10* anti-inflammatory cytokine (Fig 1C). Conversely, after low iron diet liver *Arg1* was reduced. Since hepatocytes express Arg1 [51] we further assessed the impact of iron status on macrophages in the peritoneum compartment. Peritoneal macrophages were isolated from mice receiving iron-rich or iron-deficient diet. Indeed, the gene expression of *Arg1*, *Ym1*, *IL-10* and *Stat6*, a series of genes expressed in M2 polarized macrophages, was increased after high iron diet condition, and, conversely, the expression of *Arg1* and *IL-10* was significantly decreased after dietary iron deficiency (Fig 1D). Thus, *in vivo* dietary-induced modifications of iron levels influence the expression of type 2 immune response markers.

**Fig 2:**
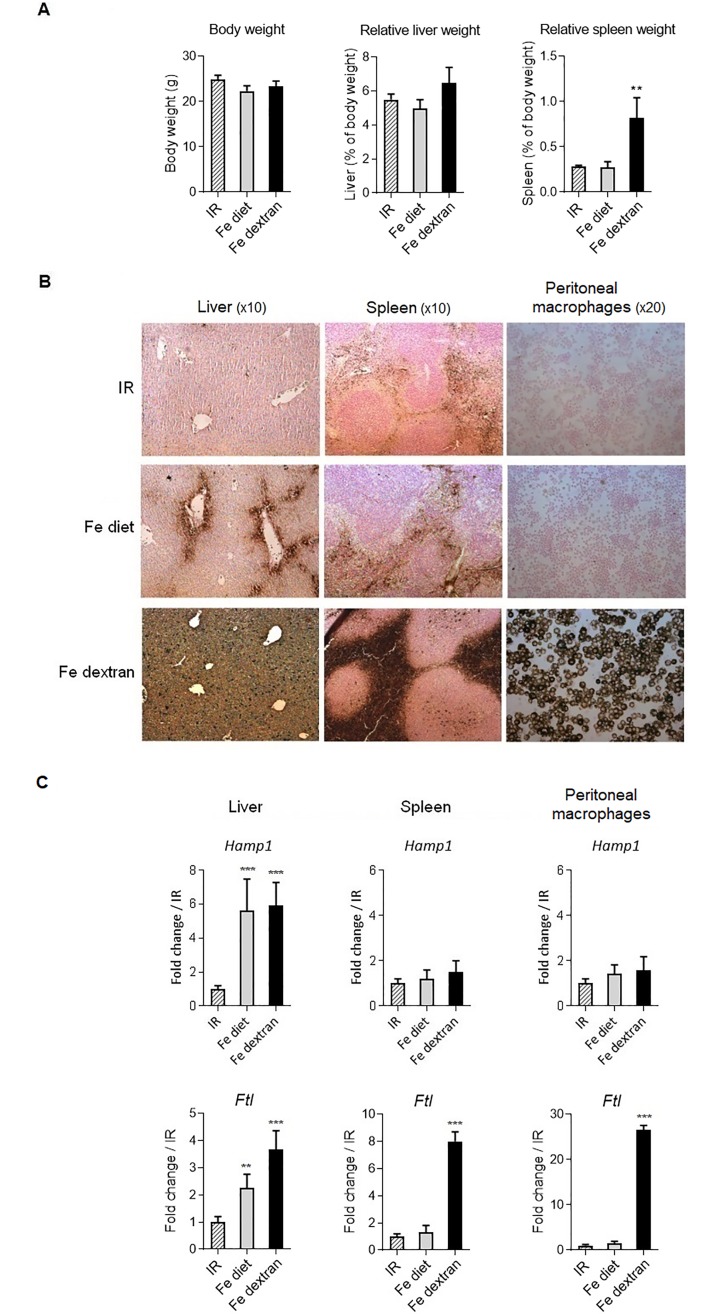
Iron status in liver, spleen and peritoneal macrophages. C57BL/6 wild type mice were fed with iron replete diet (IR), or iron-rich diet for 3 days (Fe diet) or received an injection of iron-dextran (Fe-dextran, 0.2 g/kg ip) for 48h. (**A**) Body weight, relative liver and spleen weights are indicated. (**B**) Representative DAB-enhanced Perl’s staining of liver, spleen, and peritoneal exudate cells spin show iron deposit (in brown). (**C**) *Hepcidin 1 (Hamp1*) and *ferritin L* (*Ftl*) gene expression in liver tissue homogenates, spleen and peritoneal cells was analyzed using quantitative PCR. Data are expressed as mRNA fold change relative to control mice fed with IR diet. Data are from two independent experiments and presented as mean ± SD (n = 6 mice per group). ns, non significant; * p≤ 0.05; ** p≤ 0.01; *** p≤ 0.001.

We documented the iron loading induced by feeding with a high iron-containing diet (25 g/kg) for 3 days and compared it with a systemic injection of iron-dextran (0.2 g/kg). Although overall body weight were barely affected liver relative weight, and furthermore spleen relative weight were increased after iron-dextran treatment (Fig 2A). Iron-dextran intraperitoneal injection resulted at 48h in high iron deposition in liver, including hepatocytes and macrophages, in spleen red pulp, and in cells from peritoneal exudates (Fig 2B), associated with 120-fold increased hepatic iron concentration (HIC) and elevated transferrin saturation (Table 1). The digestive route of iron loading resulted mainly in iron deposition in periportal hepatocytes (Fig 2B), and was associated with a more moderate increase in hepatic iron content and transferrin saturation (Table 1), as compared to control mice fed with iron-replete diet (IR). Spleen iron staining was equivalent for both conditions, with no iron staining of peritoneal cells (Fig 2B). The level of *hepcidin 1* gene upregulation was similarly increased in the liver of mice fed with iron-rich diet or injected with iron-dextran. *Hepcidin 1* gene expression was not altered in spleen and peritoneal cells in response to either iron treatments (Fig 2C). The expression of *ferritin L* gene, encoding the predominant subunit of ferritin in liver and spleen [52], was increased in liver under both iron overload conditions, while it was strongly enhanced in spleen and peritoneal cells after iron-dextran systemic injection, but was not affected after iron rich diet regimen (Fig 2C). The transcription of *ferritin L* gene is induced in response to oxidative challenge [53, 54] independently of iron level. Here, *ferritin L* gene overexpression was indicative of an increased ROS formation in correlation with tissue iron level. In blood, transferrin saturation percentage was increased under both iron overload conditions, whereas serum iron concentration was highly increased after iron-dextran injection and less so after iron-rich diet, as compared to mice fed with iron-replete diet (Table 1). Erythrocyte markers, including red blood cell counts, mean corpuscular volume, and hemoglobin were not affected by either iron overload conditions, but hematocrit tended to increase in iron loaded mice (Table 1). Thus, both iron-rich diet and iron-dextran injection led to liver iron overload and *hepcidin 1* upregulation, while iron-dextran also lead to systemic elevated iron deposits in spleen and peritoneal cells.

**Table 1:** For each group of mice hepatic iron content and iron blood parameters, including serum iron and percentage of transferrin saturation, as well as red blood cell counts (RBC), mean corpuscular volume (MCV), hematocrit (Ht) and hemoglobin (Hb) are indicated. Data are from two independent experiments and presented as mean ± SD (n = 6 mice per group).

Parameters	IR	Fe diet	Fe dextran
Iron			
HIC (ng Fe/mg dried tissue)	183.46 ±23.32	259.86 ±46.67	22681.22 ±9288.3 ***
SI (μg Fe/dL)	108.42 ±20	177.86 ±19.26	340.50 ±67.76 ***
Transferrin saturation (%)	47.45 ±4.50	76.73 ±7.22 **	77.87 ±15.20 **
Erythrocytes			
RBC (10^6^/μL)	8.55 ±1.84	9.36 ±0.9	9.44 ±0.68
MCV (fl)	49.44 ±8.6	46.8 ±1.45	49.8 ±2.87
Ht (%)	40.95 ±5.57	43.72 ±3.69	46.8 ±1.62
Hb (g/dL)	13.3 ±0.77	13.84 ±0.77	14.7 ±0.67

* p≤ 0.05.

** p≤ 0.01.

*** p≤ 0.001.

**Fig 3:**
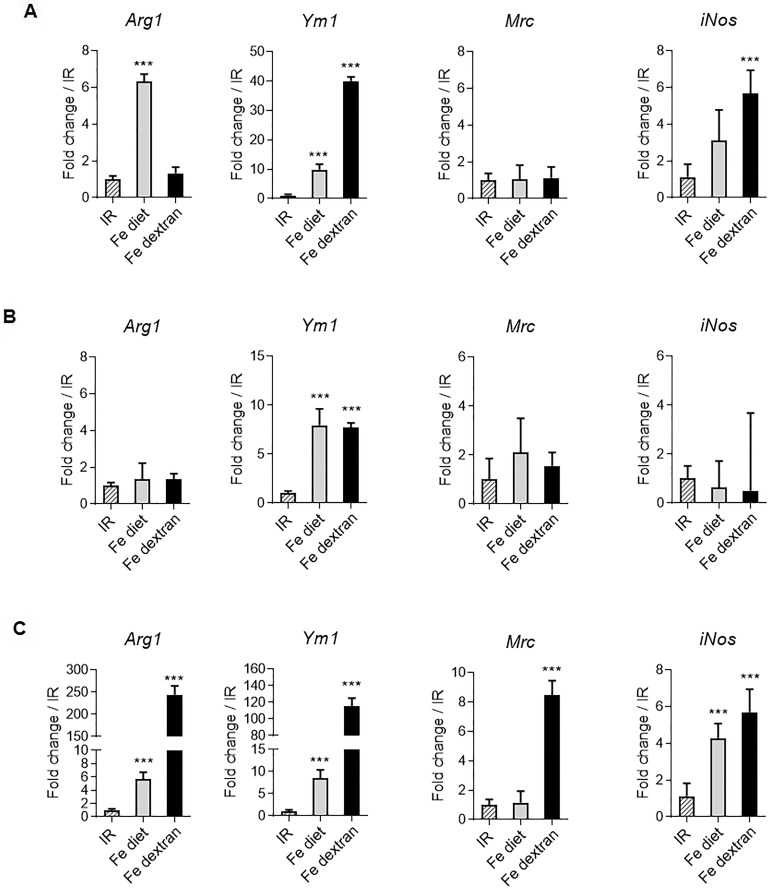
Expression of macrophage polarization markers upon iron overload in liver, spleen and peritoneal macrophages. C57BL/6 wild type mice were fed with iron replete diet (IR), or iron-rich diet for 3 days (Fe diet) or received an injection of iron-dextran (Fe-dextran, 0.2 g/kg ip) for 48h. Liver homogenates (**A**), spleen (**B**) and peritoneal exudate macrophages (**C**) gene expression of macrophage polarization marker *Arginase-1 (Arg1)*, *Chitinase-like 3 (Ym1)*, CD206 mannose receptor (*Mrc*) or *iNos* was analyzed using quantitative PCR analysis. Data are expressed as mRNA fold change relative to control mice fed with IR diet. Data are from two independent experiments and presented as mean ± SD (n = 6 mice per group). ns, non significant; * p≤ 0.05; ** p≤ 0.01; *** p≤ 0.001.

We next further characterized how iron overloading influenced markers of immune response. Hence, the gene expression of a series of markers expressed in M2 (*Arginase-1*, *Chitinase-like 3*, mannose receptor) or M1 (*iNos*) polarized macrophages was assessed in liver, spleen and peritoneal macrophages. Interestingly, in mice fed with high iron diet the expression of type 2 immune response markers *Arginase-1* (*Arg1*) and *Chitinase-like 3 (Ym1)* was increased in liver (Fig 3A), spleen (Fig 3B) and peritoneal macrophages (Fig 3C), yet *Mrc* was not affected and *iNos* expression did not change significantly after iron rich diet in liver and spleen, but increased in peritoneal macrophages (Fig 3A). In mice injected with iron dextran, *Arg1*, *Ym1*, mannose receptor (CD206) as well *iNos* expression was enhanced in liver homogenate, spleen and peritoneal macrophages (Fig 3A, 3B and 3C). Thus, *in vivo* high iron levels influence the expression of both type 1 and type 2 immune response markers.

### Iron status influences the IL-12/IL-10 axis balance during endotoxin-induced inflammation in peritoneal macrophages

**Fig 4:**
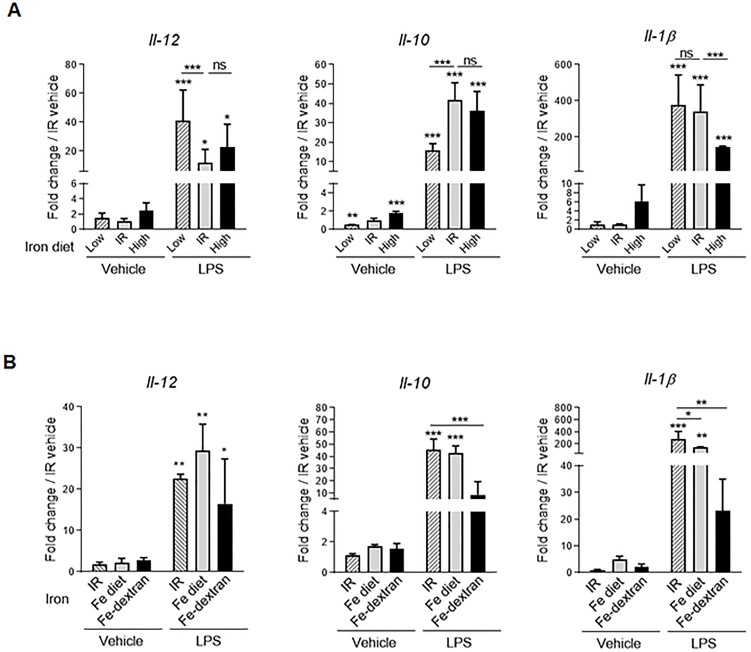
Iron deficiency increases LPS-induced IL-12/IL-10 balance *in vivo*. C57BL/6 wild type mice were either fed with iron replete diet (IR) or iron-deficient diet for 14 days (Low) or iron-rich diet for 3 days (High), or treated with iron-dextran (Fe-dextran, 0.2 g/kg ip) for 48h. Mice were then treated with LPS (i.p 50 μg/kg) or NaCl 0.9% as vehicle for 4 h. Peritoneal cells were collected to assess the *Il-12*, *Il-10*, and *Il-1β* gene expression using quantitative PCR. (A) data from mice fed with various diets and (B) comparison of mice fed with high iron diet or treated with iron-dextran. Data are expressed as mRNA fold change relative to vehicle control mice under IR diet. Gene expression was then analyzed using quantitative PCR analysis. Data are expressed as mRNA fold change relative to control mice fed with IR diet. Data are from two independent experiments and presented as mean ± SD (n = 6 mice per group). ns, non significant; ** p≤ 0.01; *** p≤ 0.001.

Our results suggested that *in vivo* diet-induced iron status impacted immune polarization. We next hypothesized that changes of iron status could alter the inflammation process in macrophages. We thus assessed the effect of iron diet on lipopolysaccharide (LPS)-induced pro-inflammatory response in peritoneal macrophages. Adult C57BL/6 mice fed with high or low iron diet were treated with a sublethal concentration of LPS (50 μg/kg) or vehicle for 4 hours and cytokine gene expression in peritoneal cells was determined. In naïve cells from mice fed with either high or low iron diet *Il-12* and *Il-1β* gene expression were not affected as compared to iron replete, while *Il-10* was influenced by the iron diet (Fig 4A). In response to LPS treatment, the expression of *Il-12*, *Il-10* and *Il-1β* was clearly modulated by the iron status (Fig 4A). Iron deficiency promoted LPS-induced *Il-12* gene expression and decreased *Il-10* gene expression in response to LPS, while *Il-1β* was significantly reduced after iron supplementation (Fig 4A). *In vivo* extreme iron loading after iron-dextran injection yielded a decrease in *Il-1β* and *Il-10* (Fig 4B). Thus, *in vivo* iron status modulates the immune responses, dietary iron deficiency exacerbated pro-inflammatory effects of endotoxin whereas dietary iron loading decreased some inflammatory responses such as Il-1β expression.

### Macrophages iron loading impairs IFN-γ-induced M1 polarization

**Fig 5:**
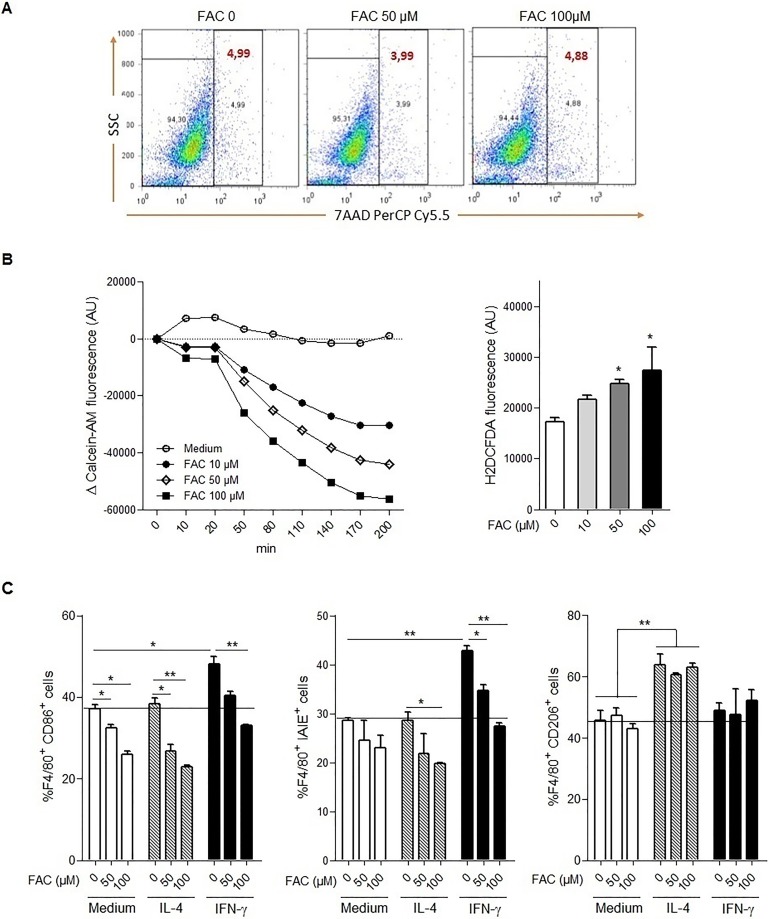
Iron loaded macrophages express constitutive M1 cell surface markers *in vitro*. **A**: Bone marrow-derived macrophages incubated in 6-wells plate were treated overnight with ferric ammonium citrate (FAC 0, 50 or 100 μM) as indicated. Thereafter, cells were stained by 7-Aminoactinomycin D (7-AAD) and membrane integrity analyzed by flow cytometry. The percentage of viable cells is given by excluding all the positive cells. **B**: Bone marrow-derived macrophages plated in 96 well microplate overnight were washed with PBS and treated with either calcein-AM (5 μM, 5 min) for labile cell iron measurement or with H2DCFDA (5 μM, 30 min) for intracellular reactive oxygen species (ROS) assessment. After addition of ferric ammonium citrate (FAC, 10, 50 or 100 μM), fluorescence measurements were performed at different time points to determine the level of labile cell iron or after 16 hours for intracellular ROS. **C**: Bone marrow-derived macrophages incubated in 6-wells plate were preincubated 2 hours with ferric ammonium citrate (FAC, 0, 50 or 100 μM), then incubated with IL-4 (10 ng/mL) or IFNγ (100 UmL) overnight. The percentage of CD86^+^, I-A/I-E^+^ and CD206^+^ cells were then assessed in F4/80^+^ cells. Data are representative of two independent experiments and presented as mean ± SD (n = 4). * p≤ 0.05; ** p≤ 0.01; *** p≤ 0.001.

To further assess the influence of iron loading on macrophage polarization, we next determined the profile of macrophages in response to cellular iron loading by using bone marrow derived macrophages incubated with ferric ammonium citrate (FAC). We first verified cell viability in response to iron loading, and showed that the FAC concentrations of 50–100 μM used in this study did not influence cell viability (Fig 5A). We then confirmed that increasing the concentration of ferric ammonium citrate in culture medium correlated with increased intracellular iron using calcein fluorescence quenching method (Fig 5B). Further, the increase of intracellular reactive oxygen species was determined using the general oxidative stress indicator 2’,7’-dichlorodihydrofluorescein diacetate (H2DCFDA). The increase in intracellular iron was correlated with an increase of reactive oxygen species, which was significantly induced after 16 hours of incubation with ferric ammonium citrate (Fig 5B). Thereafter, we determined macrophage polarization based on cell surface expression of the CD86 co-stimulatory molecules and I-A/I-E MHC class II molecules, versus CD206 mannose receptor, as markers highly expressed on M1 and M2 polarized macrophages, respectively. BMDM were gated on 7-AAD^-^, F4/80^+^ cells and analysed by flow cytometry for the membrane expression of CD86, I-A/I-E, and CD206. Interestingly, 24 hours of incubation with ferric ammonium citrate alone decreased CD86^+^ and I-A/I-E^+^ cells within the F4/80^+^ population (Fig 5C), suggesting that cell iron loading decreased the basal constitutive expression of these molecules. IFN-γ-induced CD86 and I-A/I-E over-expression was impaired by ferric ammonium citrate pretreatment and this was dose-dependent. The decrease of CD86 and I-A/I-E expression induced by iron loading was accentuated by IL-4 (Fig 5C). In contrast, the expression of CD206, increased after incubation with IL-4, as expected, was not further increased by iron loading. Thus, cell iron loading affects the expression of membrane costimulatory molecules associated with macrophage polarization, higher iron levels notably reducing M1 marker expression.

### Alteration of endotoxin-induced pro-inflammatory response by cell iron overload

**Fig 6:**
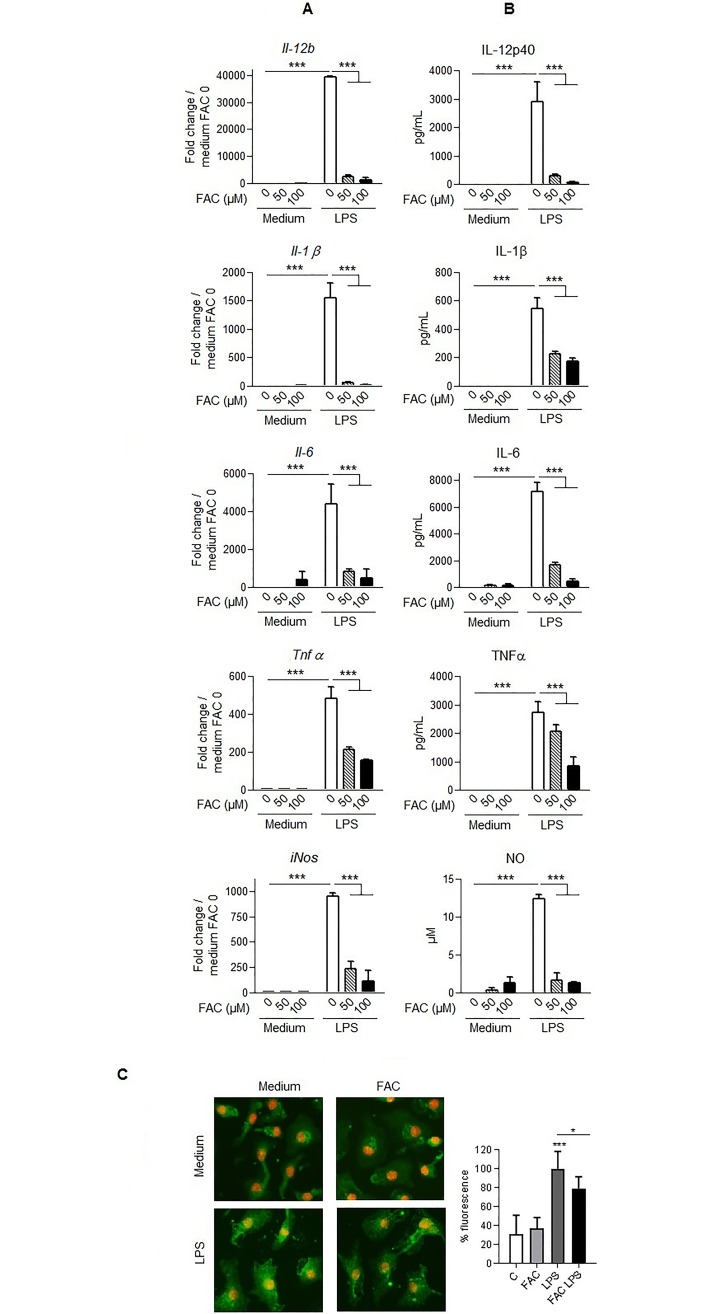
Iron loading of primary macrophages impairs LPS-induced pro-inflammatory responses. Bone marrow derived-macrophages were preincubated with ferric ammonium citrate (FAC, 0, 50 or 100 μM) overnight followed by LPS stimulation (100 ng/mL) for 4 h or 24 h to assess *Il-12b*, *Il-1β*, *Il-6*, *Tnfα* and *iNos* mRNA gene expression by quantitative PCR analysis (**A**) or cytokine Il-12p40, Il-1β, Il-6, Tnfα and nitric oxide measurement in cell medium by ELISA and Griess assay (**B**), respectively. Bone marrow derived-macrophages were preincubated with ferric ammonium citrate (FAC 100 μM) or medium overnight followed by LPS stimulation (100 ng/mL) for 30 min to assess NF-κB nuclear translocation by immunofluorescence, with NF-κB p65 in green and DAPI in red (**C**). Data of mRNA gene expression are given as fold change gene expression relative to the expression in untreated cells. Data are representative of at least two independent experiments and presented as mean ± SD (n = 4). * p≤ 0.05; ** p≤ 0.01; *** p≤ 0.001.

To further address the anti-inflammatory role of cell iron loading, we next assessed the functional consequences of iron supplementation on LPS-induced inflammation response in macrophages. BMDM were treated with ferric ammonium citrate overnight followed by TLR4 stimulation with LPS during 4 h for gene expression and 24 h for cytokine protein release and mediator analysis. The mRNA overexpression of *Il-12b*, *Il-1β*, *Il-6* and *Tnfα* genes induced by LPS (Fig 6A) was drastically reduced in BMDM pretreated with ferric ammonium citrate and this correlated with a reduction in the respective pro-inflammatory cytokines Il-12p40, Il-1β, Il-6 and Tnfα release (Fig 6B). In addition, the expression of *iNos*, a marker of M1 macrophages and its chemical product nitric oxide (NO) in response to LPS were also strongly impaired by iron loading (Fig 6A and 6B).

Early activation of TLR4 pathway leads to M1 polarization of macrophages characterized by the activation of NF-κB p65/p50 heterodimers, leading to the expression of pro-inflammatory cytokines and nitric oxide production. To assess the role of NF-κB p65/p50 in the modulation of the immune response by iron loading we evaluated the activation NF-κB p65. Macrophages were treated with ferric ammonium citrate overnight followed by TLR4 stimulation with LPS during 30 min to analyse NF-κB p65 nuclear translocation. As shown in Fig 6C, neither medium nor ferric ammonium citrate incubation allowed NF-κB p65 nuclear translocation, whereas LPS treatment on macrophages led to translocation of NF-κB p65. Interestingly, pretreatment of macrophages with 100 μM of ferric ammonium citrate impaired the LPS-induced nuclear translocation of NF-κB p65 (≈21%). Thus, *in vitro* cellular iron loading reduced NF-κB p65 nuclear translocation, *iNos* and pro-inflammatory cytokines expression, indicating that iron overload directly prevents macrophage proinflammatory response to endotoxin.

### Intracellular iron load differentially modulates macrophage *hepcidin* and *ferroportin* expression in response to LPS

**Fig 7:**
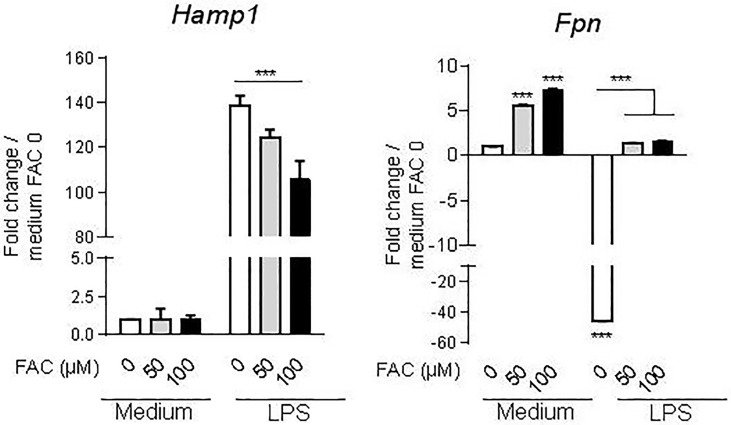
Iron loading modulates LPS-induced *hepcidin 1* and *ferroportin* expression in macrophages. Bone marrow derived-macrophages were incubated with ferric ammonium citrate (FAC, 0, 50 or 100 μM) for 16 hours followed by LPS stimulation (10 ng/mL). *Hepcidin 1* (*Hamp1*) and *ferroportin* (*Fpn*) mRNA expression were determined after 4 h (*Fpn*) or 12 h (*Hamp1*) using quantitative PCR analysis. Data are presented as fold change gene expression relative to the expression in untreated cells. Data are representative of at least three independent experiments and presented as mean ± SD (n = 4). *** p≤ 0.001.

Iron-loading of macrophages impairs pro-inflammatory gene expression associated with a decrease of the expression of CD86 and I-A/I-E cell surface molecules associated with M1 polarization. Further, *hepcidin 1* upregulation and/or *ferroportin* downregulation have been associated with different inflammatory stimuli such as TLR agonists or cytokines [45, 47, 10]. Thus, we next analyzed how iron cellular loading might influence the expression of *hepcidin 1* and *ferroportin* in macrophages. As shown in Fig 7, intracellular iron loading decreased LPS-induced *hepcidin 1* mRNA expression dose-dependently while iron treatment alone had no effect. The *ferroportin* expression was increased in response to iron, and strongly downregulated after LPS exposure in macrophages. Interestingly, the LPS-induced downregulation of *ferroportin* mRNA in macrophages was abrogated by ferric ammonium pretreatment (Fig 7). Conversely, LPS stimulation prevented the upregulation of *ferroportin* in response to iron. Thus, the impaired expression of proinflammatory genes, cytokines and costimulatory receptors in response to LPS after macrophage iron overloading was associated with a reduced expression of *hepcidin 1* gene and restoration of *ferroportin* gene expression.

## Discussion

Our data show that increased cell iron loading triggers the expression of monocyte polarization markers of M2-like phenotype in resting macrophages and dampens pro-inflammatory immune responses, while iron deficiency has the opposite effect. Indeed, *in vivo* increased iron status leading to increase transferrin saturation and tissue iron deposition, stimulated expression of M2-associated markers and cytokines, such as *arginase-1*, *Ym1* chitinase-like and mannose receptor. Modifications in iron balance also altered cellular inflammatory responses, as increased iron levels repressed some type 1 immune responses to TLR4 stimulation, such as *iNos* and *Il-12* gene expression. Indeed, after TLR4 stimulation iron-loading of primary bone marrow derived macrophages repressed the expression of M1-associated markers, decreased NF-κB p65 nuclear translocation, pro-inflammatory cytokines release and NO formation, while promoting M2-associated markers. Conversely, iron deficiency promoted type 1 pro-inflammatory cytokine expression in response to TLR4 stimulation. Therefore, changes in cell iron concentration can modulate macrophage phenotype and function with clear implications for the immune responses.

The effect of iron status on macrophage is consistent with previous reports of decreased CD86 and I-A/I-E expression in bone marrow derived macrophages in response to the iron chelator deferoxamine in the absence of an inflammatory context [11], or impaired *iNos* expression and NO release, M1 associated markers, in IFNγ treated RAW264.7 monocytic cell line after iron loading [55]. Interestingly, intracellular iron dysbalance disrupted the L-arginine metabolism as a precursor for iNos and arginase-1, M1 and M2 activity, respectively [16, 21, 56]. We showed that cellular iron status influences macrophage metabolic programs, which are a key component of macrophage plasticity and polarization, instrumental to their function in homeostasis, immunity, and inflammation [16, 17, 22, 23, 26, 30]. We also document that changes in cell iron status interfere with the inflammation process by impairing LPS-induced pro-inflammatory response. The IL-12/IL-10 balance is one of the most striking features of macrophage polarization. Activated macrophages M1 and M2 produce IL-12^hi^ IL-10^lo^ and IL-12^lo^ IL-10^hi^, respectively, which are cytokines involved in their pro-inflammatory and anti-inflammatory functions [1, 16, 17]. Indeed, IL-12/IL-10 ratio in response to LPS was significantly increased under iron deficiency, suggesting a pro-inflammatory role of iron deficiency in macrophages. Conversely, iron overload tended to decrease the IL-12/IL-10 balance as well as other pro-inflammatory cytokine response. Furthermore, we showed that NF-κB p65 translocation into the nucleus was reduced in our model of iron loaded macrophages, which can account for the reduced pro-inflammatory cytokine expression. NF-kB is a redox-sensitive transcription factor, and ROS are clearly implicated in its activation [57–59]. Here, the decrease in NF-κB p65 nuclear translocation after LPS stimulation was accompanied by an increase in ROS production. High ROS production by phagocytes mediates bacteria killing, but it has been previously shown that non-pathogenic levels of ROS, such as induced by certain commensal bacteria via TLRs or by H_2_O_2_ treatment, had suppressive activity on host inflammatory pathway and dampened the innate immune response [60, 61]. Indeed, ROS trigger inactivation of redox sensitive ubiquitin ligase involved in NF-κB inhibitor IκB-α degradation, reducing nuclear translocation of the p65 subunit of NF-κB [60, 61]. The decrease in NF-κB p65 nuclear translocation evidenced in iron loaded macrophages may be a consequence of low levels of ROS formation, and result in the suppression of NF-κB pathway activity and the alteration of pro-inflammatory genes expression.

Changes in inflammatory responses according to iron status were reported, although with different results [12–15]. We showed a reduced pro-inflammatory response to LPS in mice with iron overload induced by either iron-rich diet or intraperitoneal iron-dextran. These results corroborated a previous study showing that *in vivo* iron deprivation exacerbated the production of pro-inflammatory cytokines TNFα and IL-6 in liver, spleen macrophages and serum in LPS treated mice and this was reversed after hepcidin injection [12]. Other reports revealed an exacerbation of pro-inflammatory response associated with high iron status. Indeed, a model of high dose iron-dextran intraperitoneal injection (1 g/kg) exacerbated the pro-inflammatory response to a high LPS endotoxin dose (5 mg/kg) [13]. This was associated with elevated serum iron, but no data on tissue iron load was reported. In our hands, very high iron overload after iron-dextran injection (0.5 g/kg on day 8 and 4 before necropsy) and high LPS (10 mg/kg) increased LPS-induced *iNos* and pro-inflammatory cytokines expression S1 Fig). Indeed, high iron content due to erythrophagocytosis / extravasion of erythrocytes in a model chronic venous leg ulcers, or after repeated massive iron-dextran overload (0.25 g/kg every 3 days for 21 days), induced a proinflammatory M1 macrophage population [14]. Further, local iron oxide nanoparticles treatment or systemic ferumoxytol supplementation suppressed tumor growth by inducing pro-inflammatory macrophage responses with strong oxidative bursts [15]. Thus, under these conditions, the high iron tissue deposition associated with high macrophage iron levels was pro-inflammatory. In contrast, our studies show that under dietary iron supplementation, moderate iron overload is rather dampening inflammation. Thus, depending on the level of iron overload, through systemic or enteric route, the effect on inflammation might be pro-inflammatory at extremely high doses but rather anti-inflammatory at more moderate iron overload doses. In conclusion, we show here that under physiological iron loading conditions, iron-enriched diet modifies the expression of markers of macrophage polarization and influences the inflammatory responses.

## Supporting information

S1 Fig Expression of pro-inflammatory markers in liver upon high iron dextran overload. C57BL/6 wild type mice were fed with iron replete diet (C) or received an injection of iron-dextran (Fe-dextran) 0.5 g/kg on day 8 and 4 before necropsy or dextran as vehicle (C). Mice were then treated with LPS (i.p 10 mg/kg) or NaCl 0.9% as vehicle. After 4 h liver homogenates gene expression of pro-inflammatory cytokines (*Il-6*, *Tnfα*, *Il-1β*, *Ifnγ*) and *iNos* was analyzed using quantitative PCR analysis. Data are expressed as mRNA fold change relative to control mice fed with IR diet. Data are from two independent experiments and presented as mean ± SD (n = 4 mice per group). ns, non significant; * p≤ 0.05; ** p≤ 0.01; *** p≤ 0.001.
(TIF)
