# Supplementary figures and images for: Cell iron status influences macrophage polarization
Source: PLoS One. 2018 May 17;13(5):e0196921. doi: 10.1371/journal.pone.0196921 (PMC5957380; doi:10.1371/journal.pone.0196921)

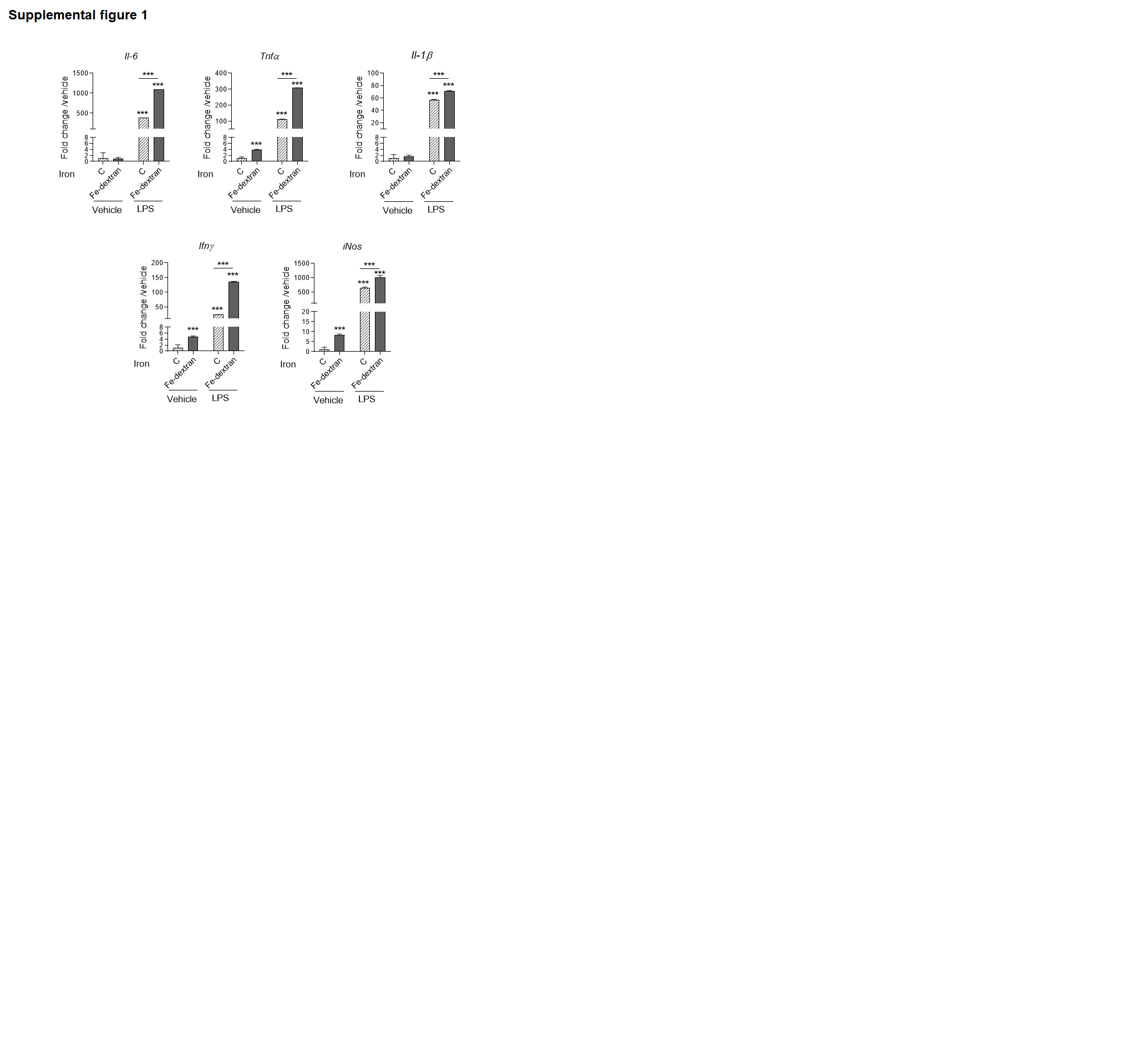

Supplement: S1 Fig — C57BL/6 wild type mice were fed with iron replete diet (C) or received an injection of iron-dextran (Fe-dextran) 0.5 g/kg on day 8 and 4 before necropsy or dextran as vehicle (C). Mice were then treated with LPS (i.p 10 mg/kg) or NaCl 0.9% as vehicle. After 4 h liver homogenates gene expression of pro-inflammatory cytokines (Il-6, Tnfα, Il-1β, Ifnγ) and iNos was analyzed using quantitative PCR analysis. Data are expressed as mRNA fold change relative to control mice fed with IR diet. Data are from two independent experiments and presented as mean ± SD (n = 4 mice per group). ns, non significant; * p≤ 0.05; ** p≤ 0.01; *** p≤ 0.001. (TIF) [file pone.0196921.s001.tif]
